# Urban Expansion of the SAFE-Home Opioid Management Education (SAFE-HOME) Naloxone Awareness Initiative for Home Health Workers and Older Adults

**DOI:** 10.3390/pharmacy9040200

**Published:** 2021-12-15

**Authors:** Abigail T. Elmes, Brianna M. McQuade, Michael Koronkowski, Erin Emery-Tiburcio, Jennie B. Jarrett

**Affiliations:** 1 Department of Pharmacy Practice, Chicago College of Pharmacy, University of Illinois, Chicago, IL 60612, USA; aelmes2@uic.edu (A.T.E.); bmcqua2@uic.edu (B.M.M.); koron@uic.edu (M.K.); 2 Division of Behavioral Sciences, Rush University Medical Center, Chicago, IL 60612, USA; erin_emerytiburcio@rush.edu

**Keywords:** naloxone, home health worker, older adult, opioid overdose

## Abstract

The SAFE-Home Opioid Management Education (SAFE-HOME) Naloxone Awareness pilot program utilized home health workers (HHWs) in rural settings to educate older adults prescribed opioids on naloxone access and use. This work expands the SAFE-HOME program to urban settings to prepare HHWs to educate community-dwelling older adults on opioid risks and life-saving naloxone. This prospective, interventional cohort study evaluated 60-min synchronous, virtual HHW educational training sessions describing opioid risks in older adults, opioid overdose signs and symptoms, and naloxone access and use. Knowledge assessments were conducted pre- and post-intervention via a pre-developed assessment tool in a repeated measure model. Outcomes included change in total opioid and naloxone knowledge, and baseline total and individual opioid and naloxone knowledge. Six educational sessions were held (*n* = 154). The average pre- and post-education scores were 62.7% (*n* = 108) and 83.5% (*n* = 82), respectively (*p* < 0.001). Of the 69 participants who completed both pre- and post-education assessments, the average change in total score was +19.6% (*p* < 0.001), opioid knowledge score −0.4% (*p* = 0.901), and naloxone knowledge score +32.9% (*p* < 0.001). At baseline, HHWs were knowledgeable on opioid risks, but lacked familiarity with naloxone access and use. Targeting HHWs with opioid and naloxone training positions them to effectively educate at-risk community-dwelling older adults.

## 1. Introduction

Older adults are often overlooked in public health efforts to address the U.S. opioid epidemic. Yet, in 2018, approximately one quarter of older adults, aged 65 years old and above, were prescribed an opioid to treat pain [1]. Older adults are nearly three times as likely to be prescribed an opioid than younger populations between 20 and 24 years old [1], potentially due to their increased frequency and severity of chronic pain [2]. Compounded with the higher use of opioids is the increased risk of serious opioid-related adverse effects in older adults which may include confusion, falls, overdose, and death. These serious risks can be exacerbated, since older adults are more likely to be taking interacting medications for other chronic health conditions or have renal or hepatic dysfunction that may increase the amount of opioids in their system [1]. Between 2016 and 2017, older adults experienced the largest increase of opioid overdose deaths in any age group (18%) [3].

Naloxone is an effective harm reduction strategy to reduce opioid overdose deaths at any age. Increasing community access to take-home naloxone can effectively mitigate opioid overdose and mortality [4]. Despite naloxone’s benefits, awareness of naloxone availability and access among older adults in the community is low [5]. On a national scale, older adults are the least likely population to obtain naloxone from a pharmacy (0.75% of older adults in 2018) [6]. The Midwest remains the lowest naloxone-distributing region in the U.S [6].

**Figure A1:**
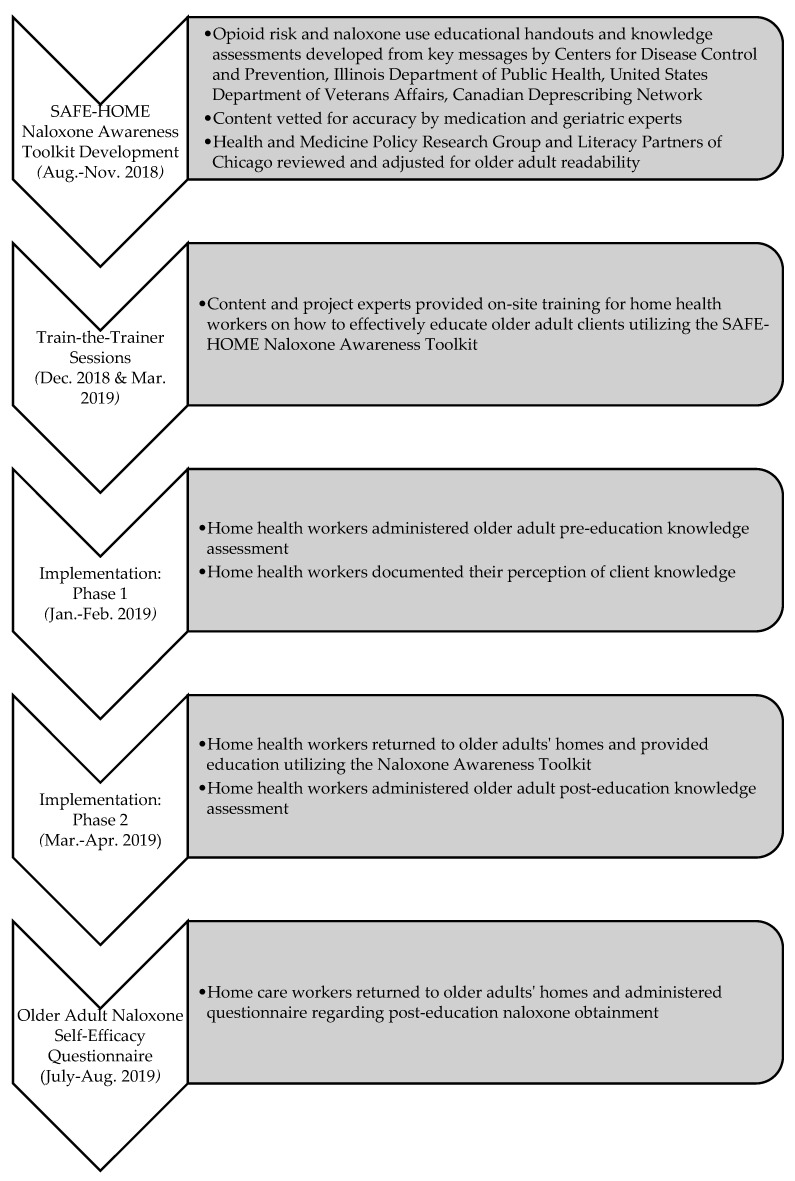
SAFE-Home Opioid Management Education (SAFE-HOME) Naloxone Awareness Initiative Pilot Program Methodology. Adapted from [5].

To address this gap, the SAFE-HOME Naloxone Awareness Initiative pilot program targeted home health workers (HHWs) in rural Illinois to educate older adults prescribed opioids on the risks of opioids and safe naloxone access and use [5]. The pilot program’s methodology is outlined in Figure A1. Program information has been previously published [5]. In brief, researchers built a Naloxone Awareness Educational Toolkit vetted for accuracy by geriatric and medication experts and for reading level and visual acuity by the Health and Medicine Policy Research Group and Literacy Partners of Chicago. In-person training sessions were held in southern Illinois to prepare HHWs to utilize the toolkit to educate older adults in their homes on opioid risks and naloxone access and use. HHWs evaluated the patient’s knowledge before and after receiving the education using a 10-question knowledge assessment. After a 4- to 8-week period, the HHWs returned to their older adult patients’ homes to repeat the knowledge assessment and assess self-efficacy, and to ascertain if, and how, the older adult obtained naloxone since receiving the education. The older adult’s knowledge (*n* = 35) at baseline was 39.4% (SD 26.8). After receiving the education, older adult knowledge increased to 90.6% (SD 12.6, *p* < 0.01), an average of 51.2%. Of the 31 older adults surveyed, 8 considered obtaining naloxone as a result of the education, but none were able to obtain naloxone, citing lack of perceived need and cost as the most common barriers [5].

This project expands the SAFE-HOME program to an urban setting and focuses on sufficiently preparing HHWs to educate older adults on opioid risks, opioid overdose, and life-saving naloxone.

## 2. Materials and Methods

This was a prospective, interventional cohort study evaluating the change in opioid and naloxone knowledge of HHWs in the Chicagoland metropolitan area after an educational intervention. Participants were recruited from the Senior Services Division of the Catholic Charities Archdiocese of Chicago (CCAC), a community-based organization that provides case coordination and care transition support for older adults, with the primary goal of maintaining independence within the home for as long as possible. HHWs regularly enter the patient’s home to provide services based on the client’s needs, including, but not limited to, navigating social security Medicare benefits, and accessing transportation, legal, and senior food services. HHWs were offered continuing education credits as incentive for participation.

In the transition to an urban setting and secondary to COVID-19 restrictions, researchers converted the HHW training session materials from the SAFE-HOME pilot program to a single synchronous, 60-min webinar format held via videoconferencing software. While the majority of the content remained the same, including a focus on opioid risks, particularly in older adults, signs and symptoms of opioid overdose, and appropriate naloxone access, product availability, and administration, updates on overdose rates and naloxone access were made. Implementation and integration of the Naloxone Awareness Toolkit into the HHWs’ current workflow was also discussed in collaboration with their organizational leadership.

Outcomes were assessed in a repeated measure model via the validated, pre-developed 10-question knowledge assessment (Table 1) [5]. The first four questions addressed knowledge on opioid risks and overdose, while the following six questions addressed naloxone use and access. Participants completed the assessments via Qualtrics^®^ survey immediately before and after the educational session.

The primary outcome was the change in total knowledge post-education as determined by the assessment score. Secondary outcomes included baseline opioid and naloxone knowledge, as well as the change in individual opioid and naloxone knowledge. Utilizing IBM SPSS Statistics for Windows, version 26 (IBM Corp., Armonk, NY, USA), descriptive statistics were used to determine average assessment scores, and paired and independent t-tests were used for paired and unpaired assessment scores, respectively, to evaluate the knowledge change pre- and post-education. Institutional Review Board (IRB) approval was obtained through the University of Illinois Chicago Office for the Protection of Research Subjects (Protocol #2018-1248).

## 3. Results

**Figure 1:**
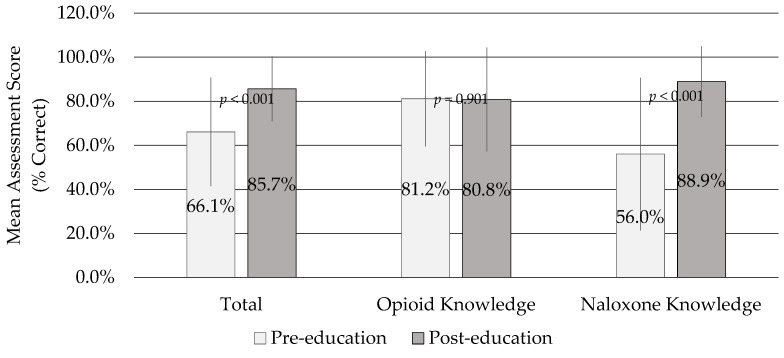
Matched Pre- and Post-Education Home Health Worker Assessment Scores (*n* = 69).

Researchers held six educational sessions across six locations of the CCAC between June and November 2020 for a total of 154 HHW participants. The response rate for completion of any knowledge assessment was 78% (*n* = 120). Sixty-nine participants (45%) completed both pre- and post-education assessments. Figure 1 demonstrates the change in assessment scores for these matched participants. The average change in total score was +19.6% (95% CI 13.7 to 25.4, *p* < 0.001), opioid knowledge score −0.4% (95% CI −6.1 to 5.4, *p* = 0.901), and naloxone knowledge score +32.9% (95% CI 24.6 to 41.1, *p* < 0.001).

Table 2 demonstrates the average knowledge scores for all assessments, including those unmatched. The average baseline opioid knowledge score (*n* = 108) was 75.5% (SD 26.2) and naloxone knowledge score was 54.2% (SD 34.3). For these unmatched participants, the average change in total score was +20.9% (95% CI 14.3 to 27.4, *p* < 0.001), opioid knowledge score +4.1% (95% CI, −3.3 to 1.2, *p* = 0.27), and naloxone knowledge score +32.0% (95% CI 23.5 to 40.5, *p* < 0.001).

## 4. Discussion

In this article, we present information on the expansion of the SAFE-HOME Naloxone Awareness Initiative to an urban setting. Our results show virtual educational sessions can reinforce knowledge on opioid risks and significantly increase knowledge surrounding appropriate naloxone access and use amongst HHWs caring for community-dwelling older adults. This change in naloxone knowledge was the primary contributor to the increase in total assessment score in this cohort, as the opioid knowledge scores changed insignificantly. These results align with previously published studies demonstrating improved knowledge following naloxone training [5,7].

At baseline, HHWs were relatively knowledgeable on opioid risks, but lacked familiarity with naloxone access, use, and legal ramifications. Our results indicate that while HHWs are knowledgeable about opioids and their surrounding risks, stronger emphasis on naloxone’s benefits and opportunities to access naloxone is needed. Despite low naloxone distribution across the US, an individual may obtain naloxone without a prescription in some capacity in all 50 states, 33 of which hold a statewide standing order [8]. Fourteen states hold various coverage requirements for some naloxone formulation for private health insurers and/or Medicaid [8]. Naloxone educational efforts not only improve knowledge surrounding naloxone’s benefits, but also increase a person’s willingness to take action in the event of an overdose, thereby reducing opioid overdose rates in the community [7,9]. By understanding the benefits and appropriate naloxone access and use, HHWs are better positioned to extend this education to their older adult clients and help them navigate the health system to gain access to this life-saving medication. HHWs can work with this often-overlooked population directly to reduce stigma, increase naloxone distribution, and reduce opioid overdose rates.

There are limitations to this study. The post-education assessments were obtained immediately after the educational sessions. As such, changes in assessment scores likely weighted towards recall and may limit the conclusion of increased knowledge. Additional assessments after a washout period could be utilized to assess retention of information over time. Similar to many research initiatives, the COVID-19 pandemic caused significant obstacles for ongoing, standardized SAFE-HOME project delivery and implementation. For example, this urban expansion included only HHW assessment scores from the initial training session due to shifts in their practice and interactions with their clients away from face-to-face encounters towards remote telehealth modalities. In contrast, the pilot program evaluated the utilization of HHWs for extending this education to in-home, community-dwelling older adults. While the toolkit is available electronically, technological literacy and/or access is challenging in the older adult population, thereby limiting educational implementation. Future research should be pursued to address these pandemic-related challenges, such as adapting the HHW workflow to allow for telephonic educational delivery. Differences between the utility of targeting HHWs to educate older adults in rural versus urban settings should also be explored. Despite these limitations, this project demonstrates the need to train HHWs on the risks of opioids and benefits of naloxone, in order to extend this knowledge to an at-risk vulnerable patient population.

## 5. Conclusions

Providing educational sessions to HHWs effectively reinforces their knowledge on opioids and increases their knowledge on naloxone access and use in preparation for educating their older adult patients in the community.

## Figures and Tables

**Table 1:** Home Health Worker Opioid and Naloxone Knowledge Assessment.

Assessment Item	Correct Response (True or False)
Opioid medications are safe for long-term use.	F
2. Only opioids can help chronic pain in patients.	F
3. Patient can’t overdose on opioid medication if they follow the doctor’s instructions.	F
4. Someone who is overdosing on opioids might: have trouble breathing, become unconscious and have trouble waking up, or have very small pupils (the black part in the middle of the eye).	T
5. Naloxone is a medicine that reverses the effects of opioids. It can be used if you think someone might be experiencing an opioid overdose.	T
6. Naloxone is easy-to-use and sprayed into the nose of a person who is unconscious from an opioid overdose.	T
7. Naloxone can help people who are overdosing on prescription medicines and illegal drugs like heroin.	T
8. A person must have a prescription to get naloxone from a pharmacy.	F
9. If someone is given naloxone, 911 should be called immediately for medical help.	T
10. It isn’t a crime to give someone naloxone if you think they might be overdosing from opioids.	T

**Table 2:** Unmatched Pre- and Post-Education Home Health Worker Knowledge Assessment Scores.

Average Score	Pre-Education (*n* = 108), % Correct (SD)	Post-Education (*n* = 82), % Correct (SD)	Score Difference
Total	62.7 (25.5)	83.5 (18.6)	+20.9% (*p* < 0.001)
Opioid Knowledge ^1^	75.5 (26.2)	79.6 (24.9)	+4.1% (*p* = 0.27)
Naloxone Knowledge ^2^	54.2 (34.3)	86.2 (21.1)	+32.0% (*p* < 0.001)

^1^ Opioid Knowledge Score is the average assessment score for questions 1 through 4. ^2^ Naloxone Knowledge Score is the average assessment score for questions 5 through 10.
